# Tumeur étendue de la base du crâne: adénocarcinome du sinus sphénoïdal

**DOI:** 10.11604/pamj.2017.28.297.13929

**Published:** 2017-12-06

**Authors:** Souha Kallel, Moncef Sellami

**Affiliations:** 1 Service ORL et Chirurgie Cervico-faciale, CHU Habib Bourguiba, Sfax, Tunisie

**Keywords:** Adénocarcinome, base de crane, sphénoïde, Adenocarcinoma, skull base, sphenoid

## Abstract

We report a rare case of adenocarcinoma of the sphenoid sinus manifesting as extended skull base tumor. The patient included in the study was a 42-year old woman presenting with unilateral right symptomatology consisting of nasal obstruction, diplopia and hemifacial neuralgias. Clinical examination showed paralysis of the cranial nerve pairs V and VI. Brain scanner showed voluminous heterogeneous sphenoid and clival mass reaching the right cavernous sinus, with a peripheral tissue component at the level of the sphenoid sinus. Biopsy was performed under general anesthesia, through endonasal sphenoidotomy approach. Histological examination showed non-intestinal adenocarcinoma. The patient died due to impaired general condition occurred during examinations. Skull base adenocarcinomas mainly occur in the ethmoid bone. Sphenoid origin is exceptional. Radiological appearance is not specific and suggests malignancy. Diagnosis should be suspected in patients with aggressive tumor, even when it occurs in the midline skull base.

## Image en médecine

**Figure 1:**
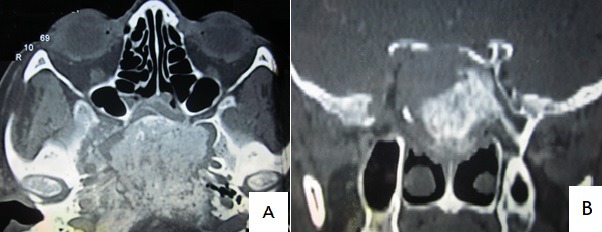
Scanner cérébral en coupes axiale (A) et coronale (B): volumineuse masse hétérogène de l’os sphénoïdal atteignant le sinus caverneux droit, avec une composante tissulaire périphérique au niveau du sinus sphénoïdal

Nous rapportons une observation rare d’un adénocarcinome du sinus sphénoïdal se présentant sous forme d’une tumeur extensive de la base du crâne. Il s’agissait d’une patiente de 42 ans qui a consulté pour une symptomatologie unilatérale droite faite d’une obstruction nasale, une diplopie et des névralgies de l’hémiface. L’examen clinique a montré une paralysie des V^ème^ et VI^ème^ paires crâniennes. Le scanner cérébral a montré une volumineuse masse hétérogène sphénoïdale et clivale atteignant le sinus caverneux droit, avec une composante tissulaire périphérique au niveau du sinus sphénoïdal. Cette dernière a été biopsiée sous anesthésie générale, par voie endonasale à travers une sphénoïdomie. L’examen histologique définitif a conclu à un adénocarcinome de type non intestinal. La patiente est décédée en altération de l’état général en cours d’exploration. Les adénocarcinomes de la base du crane prennent naissance le plus fréquemment au niveau de l’ethmoïde. L’origine sphénoïdale est exceptionnelle. L’aspect radiologique est aspécifique et évoque la malignité. C’est un diagnostic qui doit être évoqué devant une tumeur agressive, même de l’étage moyen de la base du crâne.(figure 1)

